# Graphene-Based Reinforcing Filler for Double-Layer Acrylic Coatings

**DOI:** 10.3390/ma13204499

**Published:** 2020-10-11

**Authors:** Massimo Calovi, Stefano Rossi, Flavio Deflorian, Sandra Dirè, Riccardo Ceccato

**Affiliations:** Department of Industrial Engineering, University of Trento, Via Sommarive 9, 38123 Trento, Italy; massimo.calovi@unitn.it (M.C.); flavio.deflorian@unitn.it (F.D.); sandra.dire@unitn.it (S.D.); riccardo.ceccato@unitn.it (R.C.)

**Keywords:** graphene-based filler, cataphoretic deposition process, salt spray chamber, electrochemical impedance spectroscopy, scrub abrasion test

## Abstract

This study aims to demonstrate the remarkable features of graphene-based fillers, which are able to improve the protective performance of acrylic coatings. Furthermore, the joint application of a cataphoretic primer and a spray top coat, containing graphene and functionalized graphene oxide flakes, respectively, enables the deposition of a double-layer coating with high conductivity and abrasion resistance properties, capable of offering excellent corrosion resistance to the metal substrate. The surface morphology of the single- and double-layer coatings was investigated by optical and electron microscopies, analysing the defectiveness introduced in the polymer matrix due to the filler agglomeration. The behavior in aggressive environments was assessed by exposure of the samples in the salt spray chamber, evaluating the blister formation and the adhesion level of the coatings. Electrochemical impedance spectroscopy measurements were employed to study the corrosion protection properties of the coatings, whose conductivity and abrasion resistance features were analysed by conductivity assessment and scrub tests, respectively. The incorporation of graphene-based fillers in the cataphoretic primer improves the corrosion protection properties of the system, while the graphene flakes provide the top coat spray layer with high conductivity and excellent abrasion resistance features. Thus, this work demonstrates the possibility of employing different types of graphene-based fillers and deposition methods for the creation of multifunctional coatings.

## 1. Introduction

Graphene is considered in the academic and industrial world as an innovative material, as it possesses a particular combination of physicochemical properties. As a matter of fact, the sp^2^-bonded carbon atoms, rearranged in a planar hexagonal structure [1,2], provide graphene with remarkable thermal [3,4] and electrical [5,6,7] features, as well as strong mechanical resistance properties [8,9,10]. Thus, this material is widely employed in industrial fields with high technological impact, such as the aerospace [11,12] and electronics [13,14,15,16] sectors, or bioengineering companies [17,18].

One of the applications in which graphene has found most success is the reinforcement of composite coatings [19,20,21,22], be they of metallic [23], ceramic [24,25] or polymer [26] matrix. With regard to resins, for example, graphene has shown to effectively improve the mechanical and electrical characteristics of polymeric matrices, creating multifunctional coatings [27,28,29]. However, among the various features of a coating, the main one is its effectiveness in protecting the artifact on which it is deposited. Also in this case, graphene represents a valid option as a reinforcing filler for organic coatings, enabling the realization of flame retardant barriers [30,31,32,33], wear resistant layers [34,35] and antifouling coatings [36,37].

Moreover, when added to a polymeric matrix, graphene is able to improve the corrosion resistance performance of the composite coating [38,39,40,41]. The combination of high aspect ratio and very low permeability to small molecules [42,43] allows graphene to provide a tortuous path for the absorption of aggressive species [44,45,46]. The barrier effect exerted by graphene flakes produces excellent results in the protection of metal substrates, provided that the sheets are homogeneously distributed in the polymer matrix [47], thus preventing agglomeration phenomena that would be harmful for the performance of the composite coating. Furthermore, some studies have highlighted the key role of the flakes’ spatial orientation, which influences the protective performance of the graphene-based filler [48,49,50].

Considering these latter aspects regarding the filler distribution in the resin, graphene shows significant limits. The van der Waals forces, in fact, cause the graphene sheets’ agglomeration [51,52], while the exfoliation of graphene oxide (GO) in polymeric matrix is limited by the functional groups introduced on the surface of the flakes and the hydrogen bonds [53,54]. To overcome these issues, graphene-based fillers are therefore subjected to surface conversion treatments, through functionalization processes that counteract the flakes agglomeration and improve the compatibility between graphene and polymer matrix. Among the different materials employed in the studies for the surface modification of graphene, trialkoxysilanes represent an effective option for the functionalization of this filler, due to their excellent grafting properties [40,55,56]. The silane interaction with the graphene basal plane leads to an increase of the flakes interlayer distance, improving the filler dispersion in the polymer matrix [40,57].

A recent study [58] demonstrated the high reactivity of (3-aminopropyl)trimethoxysilane (APTMS) in interacting with graphene oxide flakes. Nuclear magnetic resonance (NMR) analyses highlighted the effectiveness of the silane functionalization process, even when the sheets’ oxidation level is limited. Furthermore, the functionalized graphene oxide fillers (fGO) treated with APTMS granted excellent protective performance in acrylic matrices [47,59]. In fact, the fGO fillers caused an improvement in the corrosion resistance properties of the acrylic coating, together with a strong decrease in the permeability features of the polymer matrix. In addition, the barrier effect introduced by the flakes increased the cathodic delamination resistance of the composite coating, whose surface abrasion resistance was strengthened thanks to the high mechanical resistance features of graphene [60].

Some of the composite coatings characterized in these studies were made by means of a cataphoretic electrodeposition process, which represents a method for the realization of organic coatings with high adhesion and corrosion protection properties, in a simple and cost-effective way [61,62,63,64]. Despite the excellent features of this electrodeposition process, the literature offers few works on the addition of fillers in cataphoretic baths. However, graphene, if properly treated, has proven to be an excellent reinforcing filler, to be used also in cataphoretic processes. These works [47,59,60] paved the way for the possibility of adding different types of fillers in cataphoretic baths, exploiting their versatility.

This work is inspired by previous results [47,60], in an attempt to create multifunctional coatings, exploiting the different properties of graphene-based fillers. In this sense, we have deposited double layer coatings, each of which has particular functions and properties. The first layer, having the task of providing protective performance to the metal substrate, was made in accordance with the previous work [47]: the optimal amount of fGO (graphene oxide functionalized with APTMS) was added to the acrylic cataphoretic bath, to obtain a first coating with good protective guarantees. In the second layer, on the other hand, a large quantity of graphene has been employed, in order to increase both conductivity and abrasion resistance. Although a previous study showed the possibility of exploiting cataphoretic process for the deposition of double-layer coatings [54], the cataphoretic acrylic bath used in this work does not allow this option, as the driving force required for a second deposition cannot be supported by this type of product. Consequently, the second layer was developed using a spray deposition technique, avoiding the issues that arise in the use of graphene-based fillers in large quantities in electrodeposition processes [47]. Definitely, this work shows a novel approach for the deposition of multifunctional layers, proving the opportunity to exploit different characteristics of graphene in a single protective coating. The primer and the top coat possess specific properties, which supply the double-layer coating with unique features. A single-layer film could not exercise the same performance. Thus, the synergy of deposition processes such as cataphoresis and the spray method enables the production of a unique product, which can withstand severe chemical-physical degradation phenomena thanks to the protective contribution provided by different graphene-based fillers.

The surface morphology of the coatings and their defectiveness were assessed by observations with optical and scanning electron microscopy (SEM). The protective performances of the layers have been studied by exposing the samples in salt spray chamber and subjecting them to electrochemical impedance spectroscopy (EIS) measurements. Finally, the effect of graphene flakes on the conductivity properties of the acrylic matrix was assessed by means of selected conductivity analyses, while the abrasion behavior of the layers was characterized by scrub test.

## 2. Materials and Methods

### 2.1. Materials

Graphene powders, supplied by COMETOX s.r.l. (Milan, Italy), possess average particle diameter of about 25 µm and surface area ranging from 120 to 150 m^2^/g. Nitric acid, toluene, ethanol, acetone and (3-aminopropyl)trimethoxysilane (APTMS) were purchased from Sigma-Aldrich (St. Louis, MO, USA) and used as received. The carbon steel substrate (Q-panel type R (0.15 wt % C − Fe bal.) − 40 mm × 70 mm × 2 mm dimensions) was provided by Q-lab (Westlake, OH, USA). The acrylic-based Clear cataphoretic bath Arsonkote 202 Acy Cata W202X30 was supplied by Arsonsisi (Milan, Italy). The acrylic-based paint Smalto Acrilico 2K Lucido was provided by Palini Vernici s.r.l. (Pisogne, Italy).

### 2.2. Functionalized Graphene Oxide Filler Preparation

The cataphoretic layers were reinforced by means of functionalized graphene oxide (fGO) powder, synthetized according to the procedures performed in previous works [47,58,59,60].

Two g of graphene flakes were subjected to the oxidation process, refluxing them in 60 mL of 2.6 M nitric acid solution for 10 h. After that, the solution was allowed to cool down to room temperature under magnetic stirring, and the graphene oxide powder was separated and washed with distilled water, by means of centrifugation cycles (5 cycles of 10 min at 5000 rpm). Finally, the flakes were dried in air at ambient temperature.

Subsequently, 0.7 g of graphene oxide were mixed with 1.05 g of APTMS and 50 mL of toluene, heating the suspension to 110 °C with constant reflux for 4 h. After further 24 h of stirring at room temperature, the functionalized graphene oxide powders were filtered, washed with ethanol and dried at 50 °C for 24 h.

### 2.3. Coatings Deposition

Before the coatings deposition, the carbon steel plates were properly pre-treated, in order to favour paints adhesion. The metallic substrate was subjected to an acetone degreasing treatment, applying ultrasound stirring, followed by a sandblasting process carried out with corundum powder (0.2 mm diameter—70 mesh). Finally, a second acetone degreasing was carried out, removing the possible corundum traces from the substrate.

Regarding the layers deposited by cataphoresis process, the 200 mL acrylic cataphoretic bath was formulated following the supplier’s specifics, placing a 300 mm × 50 mm flat stainless steel anode plate at a distance of 80 cm from the sample. Two different types of coatings were made with the cataphoretic electrodeposition process. The first, called C, was deposited from the pure acrylic bath. The second, labelled CfGO, was instead reinforced introducing fGO flakes in the cataphoretic bath following the optimized conditions presented in the previous works [47,60] where the addition of 0.2 wt % of fGO in the cataphoretic bath was proven to significantly improve the protective performance of the acrylic matrix. Therefore, the fGO powder was added into the cataphoretic bath, which was stirred for 30 min with an ultrasound probe to facilitate the distribution of the graphene-based filler. During the deposition of both C and CfGO sets of samples, the process parameters have been kept constant. An applied voltage value of 75 V was used for the duration of 2 min, while the subsequent curing was carried out in oven at 140 °C for 45 min, for all the samples.

Coatings were also deposited by means of the spray technique. A layer labelled S was made with the commercial acrylic-based paint, as specified by the supplier, while SG coating was deposited by adding 1 wt % of graphene powder (G) to the acrylic paint. The coating SG was prepared with a large amount of graphene in order to achieve good conductivity properties. The resin-filler mixture was subjected to 30 min of stirring with an ultrasound probe, to effectively disperse the graphene flakes. Both the two spray layers were cured in oven at 60 °C for 60 min.

Finally, a set of samples was produced combining the previous layers. The double-layer coatings called C-SG were realized by first depositing the cataphoretic coating C, with the subsequent spray deposition of layer SG. In the coatings series called CfGO-SG, on the other hand, the SG layer was sprayed over the cataphoretic coating CfGO, containing 0.2 wt % of fGO flakes. The latter type of samples should combine the excellent corrosion resistance properties of the CfGO layer with the features of high conductivity and abrasion resistance of the SG coating. Sample labels and conditions are summarized in Table 1.

### 2.4. Characterization

The graphene-based fillers were not characterized in this work, as the same products were already widely investigated by means of optical stereomicroscope, scanning transmission electron microscope observations [60], infrared (FTIR) and X-ray diffraction (XRD) analysis [47,59] and nuclear magnetic resonance (NMR) measurements [58].

The effect of graphene-based fillers on the morphology of the protective layers has been assessed by means of optical stereomicroscope (SMZ25, Nikon Instruments, Amstelveen, The Netherlands) and low vacuum scanning electron microscope (SEM, IT 300, JEOL, Akishima, Tokyo, Japan) observations. The use of the electron microscope was also useful in defining the thickness of the individual layers, confirming the measurements made with a Surfix digital thickness gauge (Phynix, Neuss, Germany).

The samples were therefore exposed in salt spray chamber, in order to evaluate the corrosion resistance behavior of the different coatings series. The experiment was carried out for a total samples exposure of 500 h, in accordance with the ASTM B117-11 standard (5 wt % sodium chloride solution) [65]. The blister evolution, related to water uptake phenomena and layer adhesion values, was monitored and evaluated according to the ISO 4628 standard [66]. The adhesion performances of the coatings before and after exposure in an aggressive environment were also assessed with the Cross Cut Test, following the ASTM D3359 standard [67].

The coatings’ corrosion protection was subsequently quantitatively assessed by Electrochemical Impedance Spectroscopy (EIS) measurements. The electrochemical tests were carried out with a Parstat 2273 potentiostat (Princeton Applied Research by AMETEK, Oak Ridge, TN, USA), sing the software PowerSuit ZSimpWin (version 2.40) and applying a signal of about 15 mV (peak-to-peak) amplitude in the 10^5^–10^−2^ Hz frequency range. The cell setup was composed of an Ag/AgCl reference electrode (+207 mV SHE) and a platinum counter electrode, immersed in the 3.5 wt % sodium chloride solution. The coatings’ performances were assessed by immersing the samples in test solution for a total of 500 h, with a testing area equal to 5.7 cm^2^.

The conductivity of the layers, influenced by the graphene-based filler, was assessed following the ASTM D 257-07 standard [68], as already described in a previous work [47]. Two copper electrodes (sheets of 25 mm × 5 mm × 0.01 mm) were positioned in parallel to the sample’s surface, at a distance of 15 mm. The potentiostat Parstat 2273 was used to apply 10 V between the two electrodes, employing the PowerSuit ZSimpWin software, evaluating the filler contribution on the conductivity behavior of the composite coatings.

Finally, the wet abrasion resistance of the layers was characterized by means of scrub tests. An Elcometer 1720 Abrasion and Washability Tester (Elcometer, Manchester, UK) was used, exploiting the BS EN ISO 11998 standard [69] as reference for the measurements. The coatings were subjected to abrasion steps of 200 cycles (37 cycles per minute) each, with the support of 2.5 g/L sodium *n*-dodecylbenzenesulfonate solution, to simulate wet abrasion processes. After each step of abrasion, the samples were washed with distilled water and dried in an oven at 60 °C for 30 min, in order to analyse the mass loss of the coatings (using a E/50 balance, Gibertini, Novate Milanese MI, Italy, sensibility of 0.01 mg) and evaluate their resistance to wet abrasion.

Salt spray tests, EIS measurements, conductivity analyses and scrub tests were performed on three samples for each type, in order to ensure the reproducibility of results.

## 3. Results

### 3.1. Coatings Morphology

The thickness of the coatings was measured with the thickness gauge, obtaining the results summarized in Table 2. These values represent the average of 50 measurements performed on 5 samples (10 measurements per sample) for each series. The second layer’s thickness in the samples C-SG and CfGO-SG was evaluated by difference between the thickness of the total coating and the one of the first cataphoretic layer.

The thickness values achieved by the cataphoretic process (samples C and CfGO) are in agreement with the literature [47,59,60], confirming the repeatability and precision of this deposition method. The coatings made using the spray technique (samples S and SG), on the other hand, possess greater thicknesses. The result of this deposition process depends on the operator’s ability, as well as on the number of applied coats. The SG coating does not seem to be affected by the large amount of graphene filler, as it shows a thickness value comparable to the one of layer S. Finally, the dimensions of the individual layers of the C-SG and CfGO-SG samples are in accordance with those of the other series of samples: both cataphoretic and spray deposition produced coatings with constant thickness.

**Figure 1:**
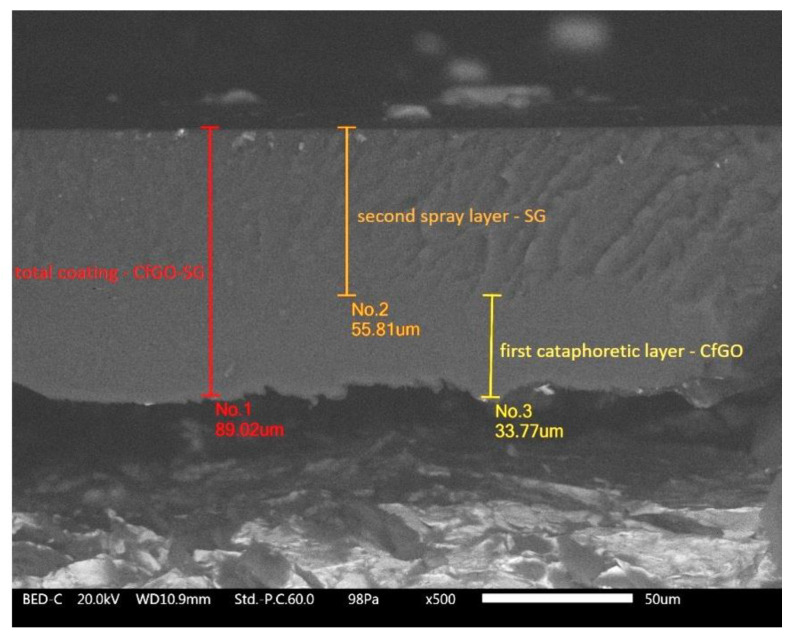
SEM micrograph of the cross section of coating CfGO-SG.

Subsequently, the samples were subjected to a fragile fracture process in liquid nitrogen, to be able to investigate the layer cross section via SEM analysis. Figure 1, which shows the CfGO-SG sample, highlights the two different cataphoretic and spray derived layers.

The coating appears compact and homogeneous in thickness. Thanks to the different fracture morphology, it is possible to clearly distinguish the first cataphoretic layer from the subsequent one obtained by spray deposition technique. While the cataphoretic layer shows a smooth and linear fracture surface, the spray coating possesses a jagged and disconnected surface. The latter complex morphology is due to the large amount of graphene fillers inside the SG layer, where the possible flakes’ agglomerations prevent the fracture of the coating from occurring in a linear way. The thickness of the two layers measured by SEM observations confirms the preliminary results obtained with the thickness gauge.

**Figure 2:**
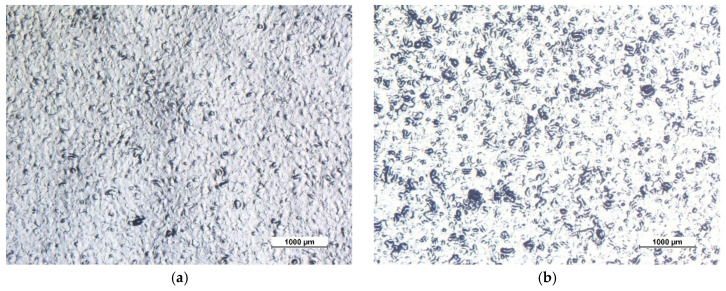

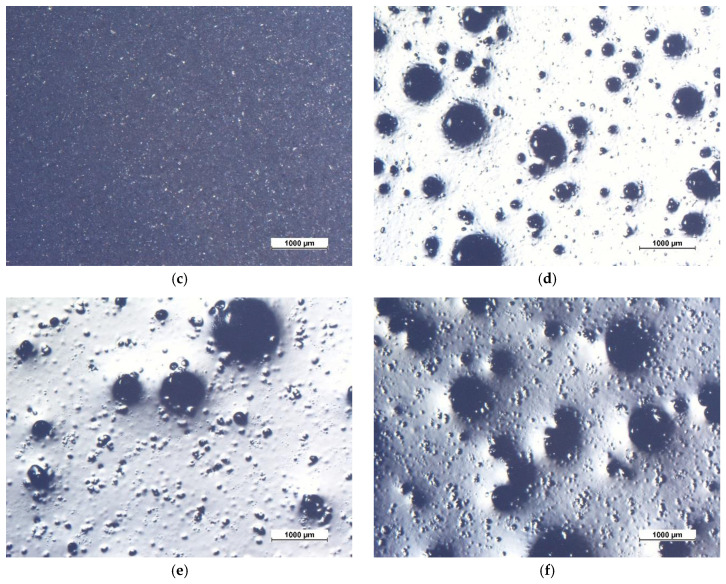
Optical stereomicroscope micrographs of the surface of sample C (**a**), sample CfGO (**b**), sample S (**c**), sample SG (**d**), sample C-SG (**e**) and sample CfGO-SG (**f**), respectively.

The presence of fillers, in addition to influencing the internal structure of the coatings, significantly alters their surface morphology. Figure 2 shows the micrographs of the six sample series, observed via an optical microscope. The two coatings obtained with the cataphoretic process, samples C and CfGO, show a low roughness surface morphology. In the coating containing 0.2 wt % of fGO there is a slight increase in porosity and bubbles, as previously observed [47,59]. The presence of the graphene-based fillers, in fact, causes the development of hydrogen bubbles during the electrodeposition process. The large amount of graphene flakes gives rise to significant changes in the morphology of the layers obtained by the spray deposition process. While the layer made only with the acrylic matrix (Figure 2c, sample S) possesses a flat surface (there is no colour contrast in the image obtained with a polarizing filter), the SG sample (Figure 2d) shows high surface irregularity due to the high tendency of graphene flakes to aggregate in polymeric matrices [51,52].

**Figure 3:**
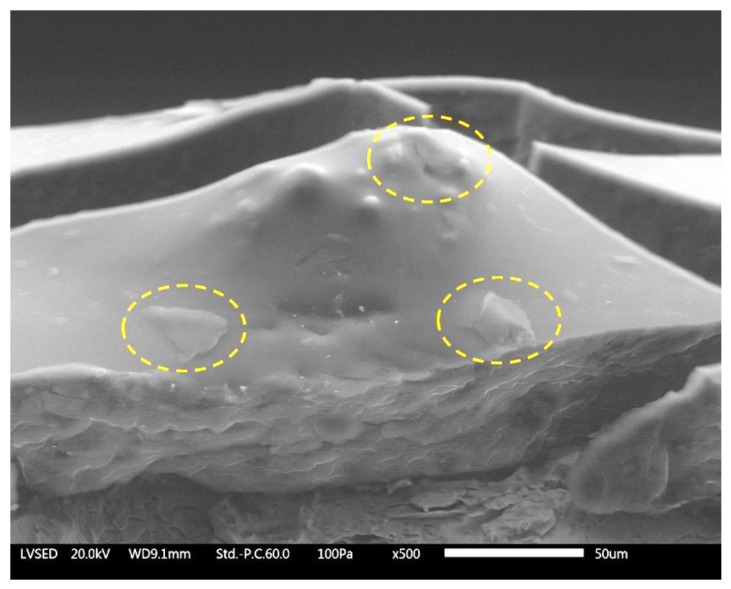
SEM micrograph of the cross section of coating SG in close proximity to agglomeration of graphene flakes.

Figure 3 shows the SEM micrograph recorded on a tilted cross section of the SG coating. Some individual graphene flakes that emerge from the surface of the layer can be clearly detected, confirming the origin of the observed surface morphology. The graphene agglomerates represent an important source of defects in the coating, but this drawback cannot be avoided when large quantities of graphene-based fillers are employed.

The same morphology is also observed in the double layer samples (Figure 2e,f), where the surface layer is the same as the SG sample (spray coating containing 1 wt % of graphene flakes). In these last two series of coatings, however, the limits due to the surface defectiveness of the SG coating should be counteracted by the first cataphoretic layer with high protective performance.

**Figure 4:**
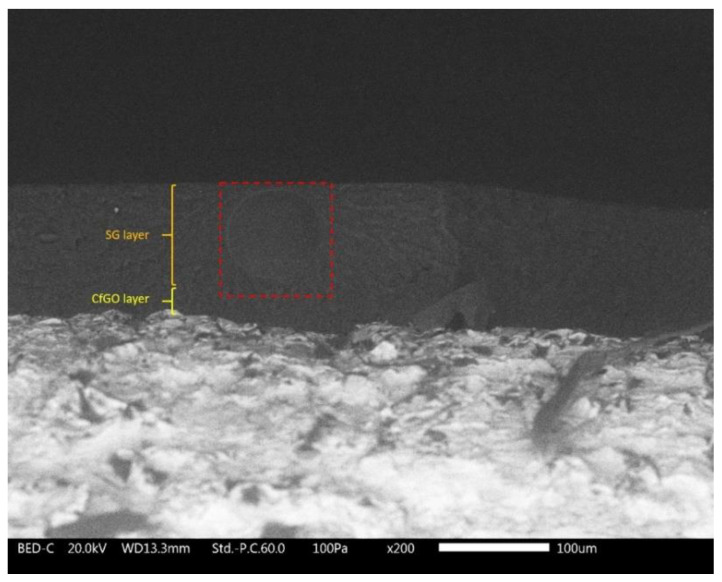
Internal porosity in the SG layer (in the red square), observed with SEM.

Nevertheless, in addition to surface accumulations, the SG layer possesses also internal defectiveness. In fact, the coatings deposited by spray technique often show a not negligible internal porosity, due to the air flow employed in the paint spray process. Figure 4 shows, as an example, the cross section of sample CfGO-SG, in which a large bubble can be observed inside the external layer (SG).

This source of defectiveness can also result in a decrease in the performance of the acrylic coatings, whose protective behavior must be adequately assessed by exposure in an aggressive environment and with electrochemical techniques.

### 3.2. Exposure in Aggressive Environment

The samples were exposed in a salt spray chamber to evaluate the protective behavior of the coatings in an aggressive environment. The degradation of the composite layers was monitored by observing the samples every 24 h, during the first 100 h of test. Subsequently, they were examined every 100 h for a total of 500 h of exposure. Moreover, a cut of 1 mm width was made on the surface of the coatings, in order to force the development of corrosive phenomena at the substrate-coating interface. Thus, the adhesion level of the layers was assessed, referring to the typical standard for sample exposure in salt spray chamber [65], and the degradation was analysed observing the development of blisters, as a consequence of the water uptake due to the artificial notch [66].

**Figure 5:**
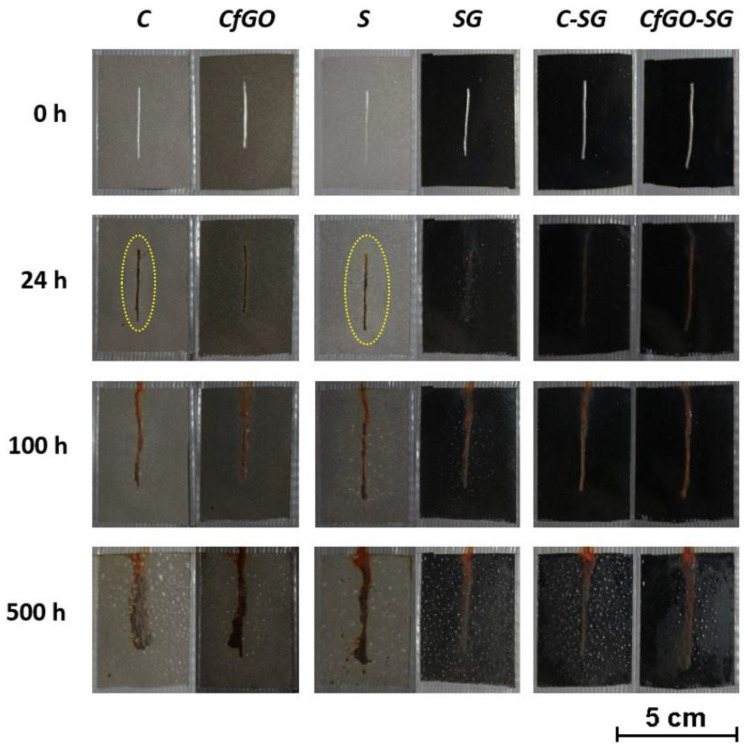
Coatings degradation during samples exposure in salt spray chamber.

Figure 5 shows the evolution of the coatings’ degradation during the salt spray chamber exposure. During the first 24 h of testing, samples C and S show an evident halo around the notch (highlighted by the yellow hatch in the image), a symptom of sudden water absorption occurrence and beginning of coating detachment. This phenomenon is less marked in the coatings containing the fillers (samples CfGO and SG), thus pointing out the protective barrier effect provided by the graphene-based flakes. These two series of samples show only a few blisters, as a consequence of the artificial defect. Double layer coatings, on the other hand, are not affected by the first 24 h in contact with the aggressive solution. The higher thickness of these coatings, in fact, provides greater protection to the substrate, with limited water absorption.

The level of defectiveness in the single layer samples increases during the first 100 h of exposure, as expected. Regarding the double-layer coatings, the first blisters appear on the surface of sample C-SG, while the CfGO-SG coating is still defects free. The latter sample, in fact, consists of two layers, the first of which (layer CfGO) was deposited to provide high protective properties, based on the results highlighted by the previous work [47]. Similarly, sample CfGO always shows a better behavior than coating C, free of filler, throughout the accelerated corrosion test.

At the end of the test, after 500 h of exposure, all the samples show evident defectiveness. The blisters cover the entire surface of the samples, except for coatings CfGO and CfGO-SG. In these two cases, the blisters developed near the artificial notch, but they did not reach the lateral edges of the samples. Once again, the layer CfGO seems to provide a good protective contribution, improving the acrylic matrix performance, both as a single coating and as a primer for the subsequent spray deposition.

**Figure 6:**
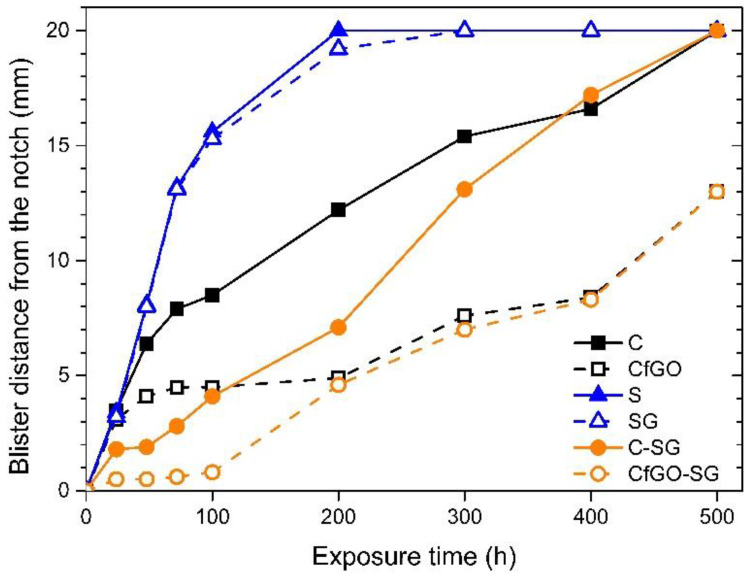
Blister development during salt spray test exposure.

The evolution of samples degradation associated with the absorption of water inside the coatings is pointed out by monitoring the blisters’ development (Figure 6). The distance from the artificial notch to which the blisters are observed is directly connected with the adhesion of the coating: the greater this distance, the greater the aggressive solution absorption inside the layer, with a consequent decrease in adhesion with the metal substrate.

Referring to the two cataphoretic layers, the sample CfGO performs better than coating C, made of pure acrylic matrix, as already highlighted in Figure 5. After 100 h of exposure there is a clear difference in performance between the two types of coating, which increases during the protraction of the test. The two spray layers display worse adhesion than the two cataphoretic coatings, with evident absorption of solution in the polymer matrix during the first 200 h of testing. This behavior is due to the spray deposition process, as the two samples S and SG have similar performances: the internal porosity shown in Figure 4 facilitates the development of blisters, as well as the accumulation of flakes in sample SG, highlighted in Figure 3. Consequently, the defectiveness of layer SG also influences the behavior of the double layer coatings. Initially, samples C-SG and CfGO-SG effectively counteract the development of blisters, thanks to the high thickness of their coatings. When, however, the SG top-layer begins to absorb large quantities of solution, the protective contribution is provided only by the cataphoretic primer. Consequently, the curves of samples C-SG and CfGO-SG tend to overlap those of the single cataphoretic layers C and CfGO, respectively. Therefore, the long-term protective contribution of the SG top coating is negligible, while the behavior of the cataphoretic primer is decisive. Thus, the fGO flakes in the cataphoretic film play a fundamental role in the protective performance of the double layer coating, ensuring a good barrier effect against the absorption of aggressive solution over time.

**Figure 7:**
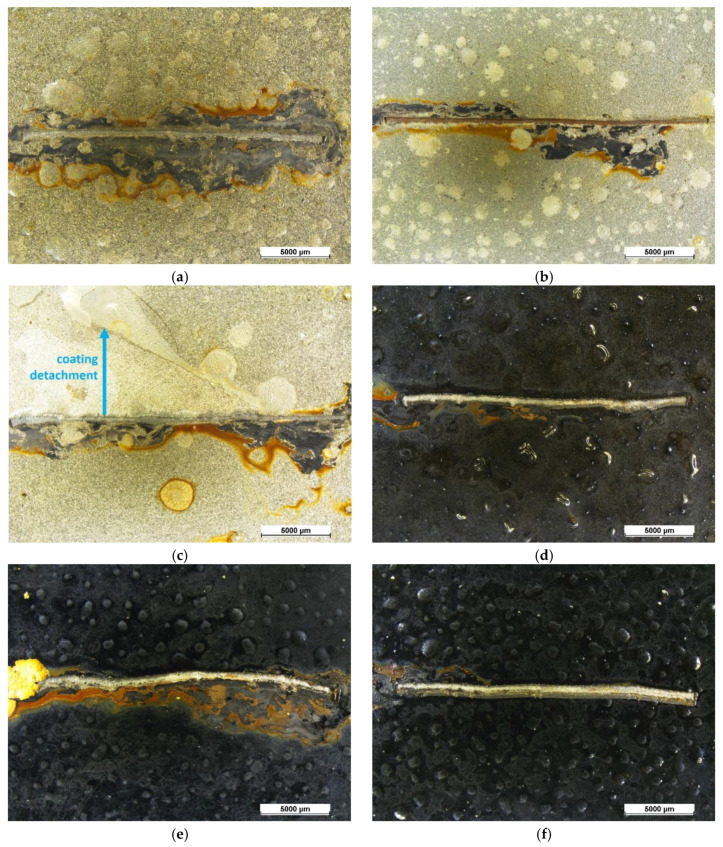
Samples C (**a**), CfGO (**b**), S (**c**), SG (**d**), C-SG (**e**) and CfGO-SG (**f**) behavior in salt spray test, after the removal of the corrosion products.

Upon removing the corrosion products by stirring of the samples in citric acid solution (pH = 3) for 2 h, the size and density of blister were evaluated by stereomicroscope observations. Figure 7 highlights the defects developed during the salt spray test. Referring to the standard [66], the single-layer coatings possess blister of size 4 and density 4 whereas the double-layer coatings show smaller blisters, of grade 2–3, with higher density, equal to level 5. According to the standard, these coatings should not be considered as performing. However, 500 h represents a very long exposure time in the salt spray chamber for an organic coating, and its degradation is expected [47,59]. Nevertheless, these results offer interesting indications. Sample C (Figure 7a) shows the development of corrosion products under the coating (dark area around the notch), due to absorption of the aggressive solution by swelling of the protective layer. This phenomenon is partially limited by the fGO flakes in sample CfGO (Figure 7b), which contrast the progress of the cathodic delamination front [60]. By comparing the two spray coatings (Figure 7c,d), the graphene fillers perform the same task, limiting the development of corrosion products below the organic layer. Finally, the protective performances of the graphene-based sheets are underlined in sample CfGO-SG (Figure 7f), which offers greater protection than the sample C-SG (Figure 7e). The comparison of Figure 7c,e with Figure 7d,f clearly highlights the ability of fGO and graphene flakes to improve the behavior of acrylic coatings in aggressive environments.

**Figure 8:**
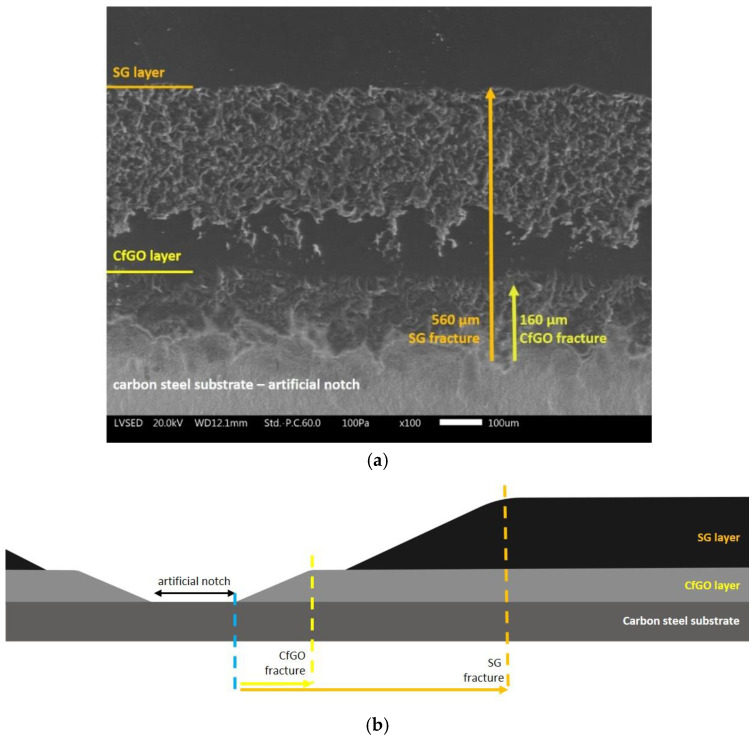
Top view of coating fracture in sample CfGO-SG, observed by SEM (**a**) and the schematic cross section of the system (**b**).

According to the standard [65], an organic coating with good adhesion to the substrate should show maximum detachment from the notch area of about 1000 μm. The corrosion that occurred below the coatings shows that these samples do not comply with this requirement. Moreover, the actual detachment of the spray layer S (shown by the arrow in Figure 7c) also occurred during the drying step of the samples by compressed air flushing, as a symptom of poor adhesion. The adhesion specification of the standard [65] was respected only by sample CfGO-SG, which did not show evident detachments of the coating due to the development of corrosion products. Figure 8a reveals the different behaviour of the two layers of sample CfGO-SG, observed by SEM in a top-view, while Figure 8b represents the schematic cross section of the system. The cataphoretic primer shows a small fracture (at the bottom in the image), equal to 160 µm in length. Differently, the volume increase of the corrosion products provoked a more acute removal of the SG top-layer (at the top of the image). However, the delamination of the coating is negligible, confirming the good adhesion properties of the coating. The adequate adhesion values of this sample is mainly due to two factors: the high thickness, which limits the absorption of the solution, and the highly protective CfGO primer layer. The combination of these two elements allows sample CfGO-SG to stand out over the other coatings, as previously highlighted in Figure 6.

**Figure 9:**
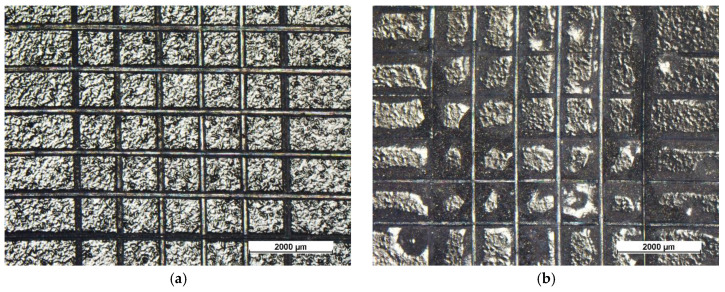
Cross Cut Test results of sample C before (**a**) and after (**b**) exposure in salt spray chamber, observed with stereomicroscope.

The adhesion features were also evaluated by Cross Cut Test, performed before and after the exposure of the samples in the salt spray chamber. Despite the different nature of the coatings, all the samples showed similar behavior. Before the salt spray test, the coatings possess good adhesion, equal to value 5B of the standard [67]. 5B is the highest degree of adhesion defined by the standard, where the edges of the cuts are completely flat and the grid shows no detachment. Differently, after 500 h of exposure in an aggressive environment, the absorption of water in the coatings caused the collapse of the adhesion values with the metal substrate. In this case, the visual results reflect grade 1B of the standard [67]. The surface of the coating that peeled off after the measurement varies between 35% and 65%. Figure 9 shows, as an example, the comparison of the test results on the surface of the coating C, before (a) and after (b) exposure in salt spray chamber.

The CfGO-SG sample showed excellent adhesion behavior in the salt spray chamber, but the Cross Cut Test highlighted that the inevitable absorption of solution in the polymer matrix causes a clear decrease in the performance of adhesion. However, the peeling of the coating occurs at the interface with the substrate, without detachment between the cataphoretic primer and the spray topcoat. Therefore, after the exposure in the salt spray chamber, the adhesion values between the two layers is good, proving a good compatibility between the primer and the top coat. The effective adhesion between the two films of sample CfGO-SG is a further justification of the superior performance of the double layer coating in an aggressive environment.

### 3.3. Electrochemical Impedance Spectroscopy Measurements

Electrochemical impedance spectroscopy represents one of the most employed techniques for the assessment of the protective performance of organic coatings. This approach provides information on defects, degree of adhesion and corrosion resistance properties of the layers [70,71].

**Figure 10:**
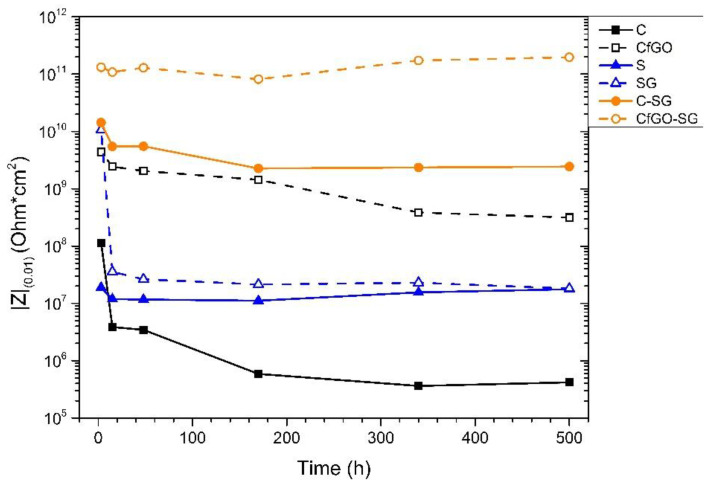
Bode impedance modulus |Z|_(0.01)_ evolution with time.

As a preliminary approach, EIS measurements produce quantitative results on the behavior of the coatings. By monitoring the evolution of the impedance module measured at low frequencies (10^−2^ Hz), defined as |Z|_(0.01)_, it is possible to get a first estimate of the degree of protection supplied by the coating. According to the literature [72,73,74], in fact, a coating must show a value of |Z|_(0.01)_ above 10^6^ Ω*cm^2^ to be able to provide adequate protective guarantees. Thus, the samples of the six series of coatings were subjected to EIS measurements, evaluating the progress of their impedance module |Z|_(0.01)_ over time. Figure 10 shows the variation of |Z|_(0.01)_ during the test.

The cataphoretic monolayer coatings show very different results. The previous work [47] had already amply demonstrated that the 0.2 wt % of fGO enables the significant improvement of the protective behavior of the acrylic matrix. While the value of |Z|_(0.01)_ of coating C falls below the protection threshold value within 200 h of immersion in the test solution, the curve of sample CfGO highlights a better corrosion resistance provided by the graphene-based filler.

Differently, the spray layers S and SG show similar behavior, with |Z|_(0.01)_ settling just above 10^7^ Ω*cm^2^ during the test. The coating SG initially shows high values of the impedance modulus, which however collapse during the first hours of immersion. The protective effect given by the high concentration of filler therefore quickly disappears, due probably to the defectiveness caused by the agglomerations of flakes, but also to the intrinsic porosity of the spray layers, which lowers the protective performance of the polymer matrix.

Finally, the double-layer coatings offer better performance than the single-layer samples. This result was expected, as the high thickness of the two coatings offers a better capacitive and resistive effect, providing higher values of |Z|_(0.01)_. After all, these two samples supply better quantitative performance than the simple sum of those of the two layers of which they are made. The synergy of the two layers, the cataphoretic primer and the spray top coat, produces a positive effect, with high protective behavior. Sample CfGO-SG shows absolutely the best results, as the primer CfGO is itself more protective than the layer C. Graphene-based fillers generally improve the protective behavior of the acrylic matrix (with the exception of layer SG where only a slight effect is observed), providing an effective barrier effect against the absorption of test solution and aggressive ions [47,60].

**Figure 11:**
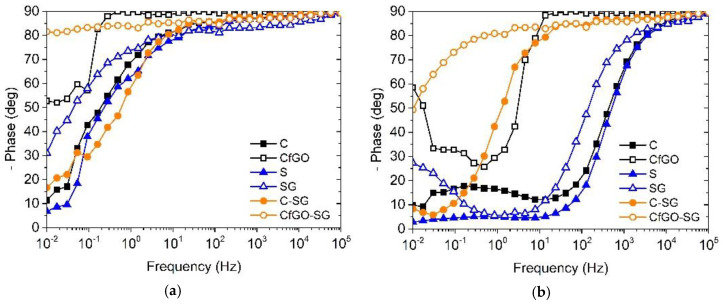
Bode phase diagrams of the samples (**a**) at the beginning and (**b**) after 24 h of immersion in test solution.

The decrease in |Z|_(0.01)_ occurs mainly during the first 24 h of immersion in the test solution. Typically, the first hours of tests are very critical for organic coatings, which can experience solution absorption phenomena. At the beginning of the measurements, the defectiveness of the layers assumes great importance. Figure 11 shows the Bode Phase diagrams of the six series of samples at the beginning (a) and after the first 24 h (b) of immersion in the test solution.

By comparing the two plots, during the EIS measurements the curves tend to shift towards high frequencies, with the simultaneous appearance of a second time constant at low frequencies. The first high-frequencies time constant is representative of the dissipation phenomena that occur through the coating, while the low-frequencies time constant is associated to the coating-substrate interface. The decrease in phase during the first 24 h of test is related to the reduction in the insulating and capacitive properties of the coating, in consequence of the absorption of solution in the coating with consequent dissipative phenomena that occur at the interface with the metal. The two-time constants curves can be fitted with the model representative of a defected layer. Each time constant is made of a constant phase element (CPE), defined as Q, in parallel with a resistance R. The resistance of the first time constant is related to the pore resistance R_pore_ of the coating, associated to the defects of the layer. On the other hand, the resistance at the low frequencies is correlated to the faradic reactions at the coating-substrate interface [75]. The CPE, in both the time constants, is employed instead of a capacitance, as the complexity of the system does not allow the correlation of non-resistive phenomena with pure capacitance. This model represents the real events occurring in the single layer coatings, enabling the data fitting with high precision and negligible errors.

Otherwise, double-layer coatings maintain high phase values, even at low frequencies. After 24 h of test, sample CfGO-SG shows a highly capacitive and insulating behavior, with a phase greater than 80 deg above 10^0^ Hz frequencies. This coating, after all, is the least affected by the immersion in the test solution, as evidenced by the high |Z|_(0.01)_ during the first hours of measurements in Figure 10. Both the two double-layer coatings possess very high impedance modulus values, higher than 10^9^ Ω*cm^2^, during the 500 h of analysis. The constancy of these two curves indicates a minimal defect in the coatings, with the absence of dissipative phenomena at the interface with the substrate. Thus, the curves were fitted with the Randles model, consisting of a single time constant made of a R in parallel with Q, representative of the degradation occurrences in the coating. The system detects the two layers as a single coating: it is therefore impossible to differentiate the defectiveness of the primer from that of the top coat, both represented by R_pore_ of the single time constant of the circuit.

Table 3 summarize the trend of the R_pore_ values of the coatings, following the fitting of the EIS curves.

The values of R_pore_ follow the same trend already shown by |Z|_(0.01)_ in Figure 10. R_pore_ remains constant over time, except for the rapid collapse in sample SG. Small increments are observed with the progress of the measurements, due to possible absorption of solution and development of corrosion products which increase the resistive effect of the system. Again, the graphene-based flakes cause an increase in R_pore_ that varies from 1 to 4 orders of magnitude respect to the corresponding filler-free coatings. This phenomenon is evident above all in the cataphoretic primer and when the graphene flakes are functionalized: the combination of chemical conversion of the filler and the type of deposition process plays a key role in limiting the defectiveness of the coating, tuning its properties. The decrease in R_pore_ slightly occurs only during the first 24 h of the test, as a symptom of the evolution of the defectiveness in the layers.

As already stated, the first 24 h of tests are the most critical for an organic coating, as its intrinsic porosity can in some cases favour the absorption of solution. The CPE of the high-frequency time constant, associated to the dissipative phenomena of the coating, can be used to calculate the amount of water uptake in the polymeric layer. Q is strictly correlated to a pure capacitance C with the formula:

C = (QR)^1/n^/R, (1)

where R represents the parameter of the resistance of the relative time constant, while n is a factor whose value allows to model the constant phase element. When n is equal to 1, the constant phase element Q can be associated to a pure capacitance. The formula expressed above has been used to calculate the values of the capacitance of the coatings during the first 24 h of exposure in the test medium. Table 4 summarizes the evolution of C during the first 24 h of EIS measurements.

**Figure 12:**
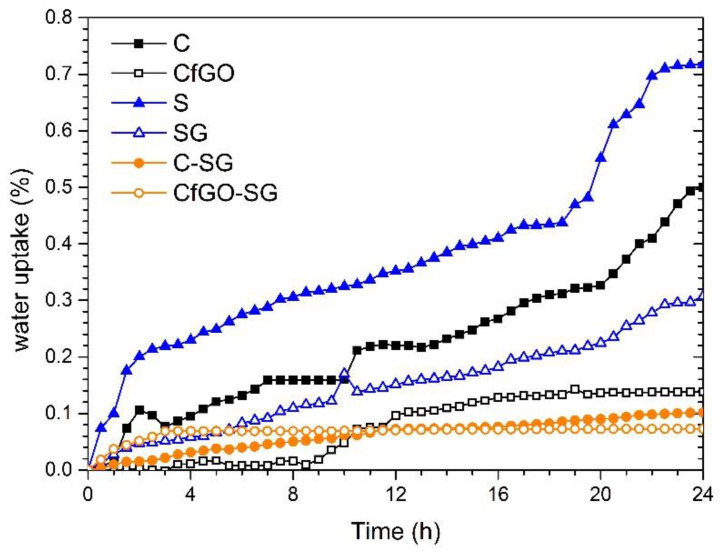
Water uptake during the first 24 h of immersion.

Thus, the values of C shown in Table 4 were employed in the calculation of the water uptake level φ, according to the formula defined by Brasher and Kingsbury in 1954 [76,77]:

φ = K(log C_t_/C_0_)/(logε_w_), (2)

where C_t_ and C_0_ represent the capacitance at an instant t and the capacitance of the “dry” coating at the beginning of the test (time = 0 h), respectively, while ε_w_ is the water dielectric constant. K is a parameter that consider the possible volume increase of the coating, but it is usually taken equal to 1. The evolution of the parameter φ is therefore shown in Figure 12.

Independently on the deposition process (i.e., cataphoresis or spray technique), graphene-based fillers confirm the excellent contribution in counteracting the absorption of solution within the composite coating. The ‘tortuous path effect’ provided by the flakes involves a decrease in the measured water uptake level, confirming the results shown in Figure 6, associated to the development of blisters during exposure in an aggressive environment. As expected, the two double-layer coatings absorb less quantity of solution, due to their greater thickness. Again, the combined behavior of the primer and the top coat is better than the sum of the performance of the two individual layers. This result confirms the good compatibility of the two coatings made with two different deposition techniques, whose responses offer high protective guarantees. Moreover, while all the samples show a continuous absorption of solution, even if limited, the coating CfGO-SG presents a very low plateau.

These electrochemical measurements evidenced that the double layer coatings, although possessing a not negligible defectiveness, offer high corrosion resistance to the metal substrate, owing to the barrier performance of the graphene-based fillers.

### 3.4. Coatings Conductivity Analysis

**Figure 13:**
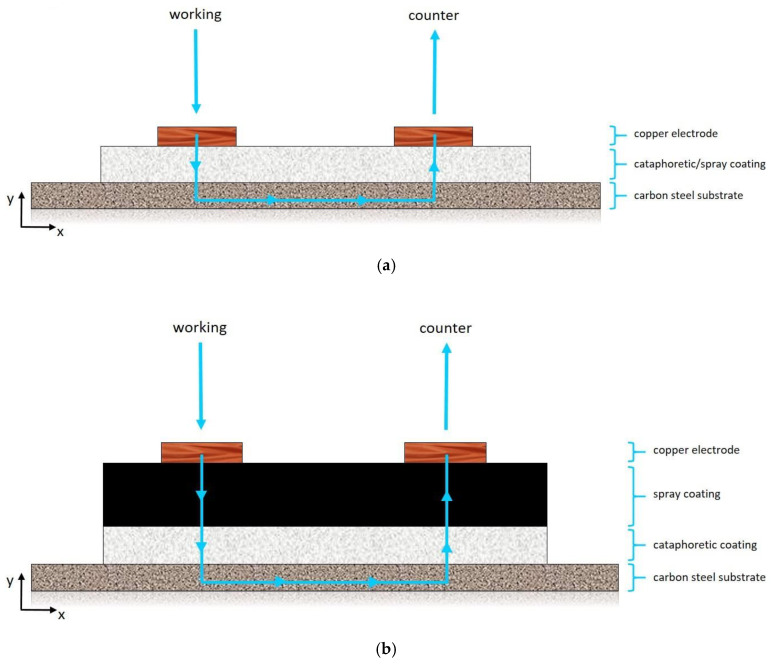
Conductivity measurement setup [cross view] for single (**a**) and double (**b**) layer coatings.

The graphene-based filler effect on the conductivity properties of the acrylic matrix was assessed according to the standard [68] already used in a similar work [47]. Figure 13 represents the test setup, which simply consists of two copper sheets used as electrodes. The approach formulated in the previous study [47] provides that during the measurement the current flow inside the coating (between the two electrodes) occurs mainly in the vertical direction (y axis in Figure 13). Thus, applying a voltage value V between the two copper sheets, the electrons departing from the working electrode can easily pass in the conductive metal substrate, and re-emerge near the counter electrode, as shown by the blue path in Figure 13a. Therefore, this procedure can be employed in the characterization of the coating resistance to the passage of charges in the vertical direction. This model has been validated for a single organic composite layer [47], but has not yet been applied to multilayer coatings. Figure 13b, for example, highlights that the high thickness of double layer coatings could represent a too long path for the electrons, limiting the conductivity along the y direction.

Table 5 summarizes the resistivity values measured during conductivity tests. The applied voltage V and the measured current I were used to calculate the Volume resistance *R*, following the first Ohm’s law. Consequently, the Volume resistivity ρ was obtained according to the formula expressed in the standard:

ρ =*R*(*A*/*t*), (3)

where *A* represents the sum of the surfaces of the two copper electrodes, while *t* is twice the thickness of the coatings (the distance that the current must travel inside the polymeric layer).

In accordance with the previous work about the optimization of the fGO content in cataphoretic coatings [47] and with the theory of the barrier effect provided by the graphene-based sheets, the Volume resistance R offered by the CfGO sample is greater than that of the coating in pure acrylic matrix (sample C). The 0.2 wt % of fGO employed in the deposition of the CfGO coating causes in fact the reduction of current passage on the y axis. The sheets, being randomly distributed inside the matrix, offer a tortuous path for the electrons, decreasing their diffusivity [50,78,79]. Thus, the Volume resistivity ρ of the CfGO coating is five times higher than the acrylic matrix, confirming the better corrosion resistance of the composite layer.

Otherwise, the fillers used in the spray layer cause a different effect. The volume resistivity ρ of sample SG is in fact less than six orders of magnitude compared to the filler-free spray coating (S). In this case, graphene flakes favour the conductivity along the y axis. This phenomenon is due to the fillers’ accumulations, well shown in Figure 3: such defectiveness allows the coating upper surface to be in direct contact with the metallic substrate. A similar result had already been observed using the same amount of fGO sheets (1 wt %) in cataphoretic coatings [47]. Such a high conductivity does not exclude that a certain amount of electrons also travels along the x axis to reach the counter electrode. Ultimately, these measurements confirm the high intrinsic conductivity of layer SG.

The conductivity performances of coating SG therefore offer an explanation of the results shown by the two double layer samples. The volume resistivity ρ values of samples C-SG and CfGO-SG are almost identical to those of the coatings C and CfGO, respectively. In fact, the double layer samples differ from the cataphoretic coatings in the addition of the second highly conductive SG layer. Since the resistive contribution of the film SG is almost negligible, the resistance to the charge motion is provided only by the first cataphoretic layer. These results confirm the high conductivity of the coating containing 1 wt % of graphene flakes, but also the validity of the model shown in Figure 13b: the current flow probably occurs along the y axis even in the two double-layer samples, since the resistivity of the coating is similar to that of the single cataphoretic layer.

Finally, these measurements revealed the multi-functionality of multilayer coatings: while the first cataphoretic film has the task of providing protection to the metal substrate, the second layer supplies high conductivity to the component, offering these coatings the possibility to be employed in electronics applications.

### 3.5. Scrub Abrasion Test

The scrub test is a technique used for the study of different features of organic coatings, such as their hydrophobic behavior [80] and antibacterial performance [81,82,83]. Recently, this approach has been employed to assess the reinforcing contribution of pigments [84] and inorganic [85] and organic [60,86] fillers in countering surface abrasion phenomena of the protective layer.

**Figure 14:**
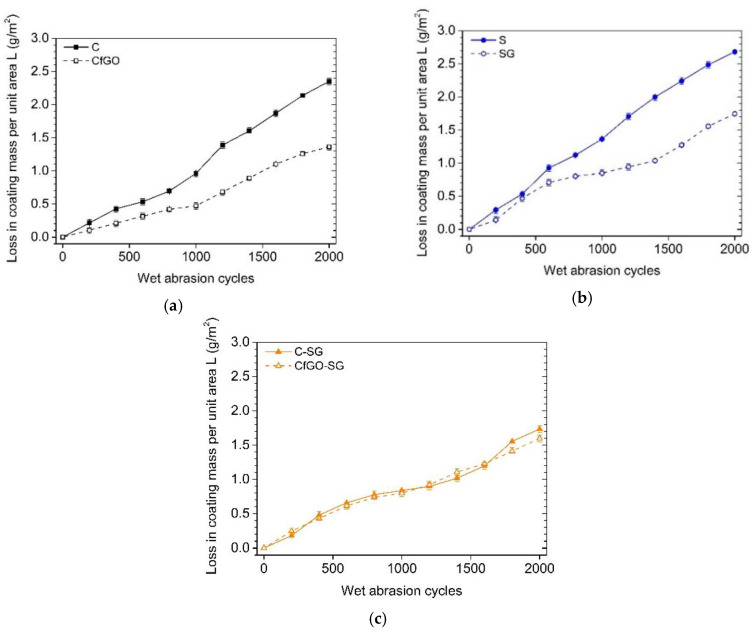
Loss in coatings mass per unit area, as a function of the abrasion cycles number, for (**a**) cataphoretic layers, (**b**) spray coatings and (**c**) double layer samples.

Following the standard [69], the wet abrasion resistance of the coatings was evaluated with a particular abrasive pad (30 mm × 80 mm × 10 mm), in association with 2.5 g/L sodium *n*-dodecylbenzenesulfonate solution. The pad, performing several abrasion cycles (37 cycles per minute), causes the removal of material on the surface of the coating. The weight loss of the samples was monitored every 200 cycles, until reaching 2000 total cycles, to estimate the contribution of the graphene-based fillers. The parameter L, defined as the loss in coating mass per unit area, was calculated with the formula:

L = (*m*_0_ − *m_n_*)/A, (4)

where *m*_0_ and *m_n_* represent the sample’s initial weight and the weight after the nth cycle, respectively, and A is the area traversed by the scrub pad over the coating’s surface. Figure 14 shows the trend of the mass loss as a function of the abrasion cycles number.

Figure 14a,b highlight the reinforcing contribution of the graphene-based fillers in the cataphoretic and spray coatings, respectively. At the end of the test, after 2000 abrasion cycles, the fGO flakes cause a 42% decrease in mass loss compared to the pure acrylic matrix coating (sample C). Similarly, the graphene fillers in coating SG improve the abrasion resistance of the spray layer by 35%. These results are in agreement with the outcome of the previous work [60], which had emphasized the graphene-based filler behavior in contrasting the grinding phenomena due to the passage of the abrasive pad. The mass loss of samples CfGO and SG appears to slow in the first 1000 cycles, followed by a further increase with the perpetuation of the test. This behavior is a symptom of an initial self-lubricating contribution of the graphene-based flakes, which are exposed due to superficial removal of the polymeric material. Subsequently, the partial withdraw of filler causes an increase in weight loss trend.

**Figure 15:**
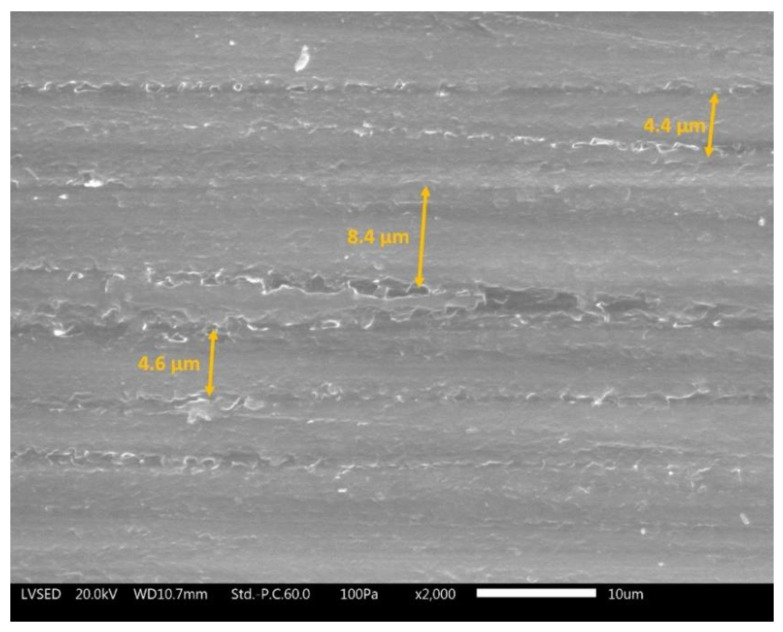
SEM micrographs of sample SG surface morphology after the 2000 scrub test cycles.

The double layer samples (Figure 14c) show almost identical results, comparable with the curve expressed by coating SG in Figure 14b. Thus, the abrasive phenomena affect only the top coat (i.e., the SG layer) which is identical for both double-layer coatings. Although the graphs in Figure 14 exhibit non-negligible weight loss values, the 2000 abrasion cycles did not involve the removal of the entire spray top-layer. As an example, Figure 15 illustrates the surface of sample SG observed with SEM, after 2000 scrub test cycles. The morphology of the coating highlights clear traces of abrasion due to rubbing with the abrasive pad. These parallel grooves possess width not exceeding 10 µm. Similarly, it can be deduced that the depth of the grooves is also less than 10 µm. The abrasive pad, despite having homogeneously removed part of the coating of the samples, did not cause a mechanical attack of particularly high depth. Consequently, the observations of the samples after scrub test did not evidence the exposure of the primer, or of the metallic substrate in the case of single-layer samples. The damage from abrasive attack can therefore be considered purely superficial.

**Figure 16:**
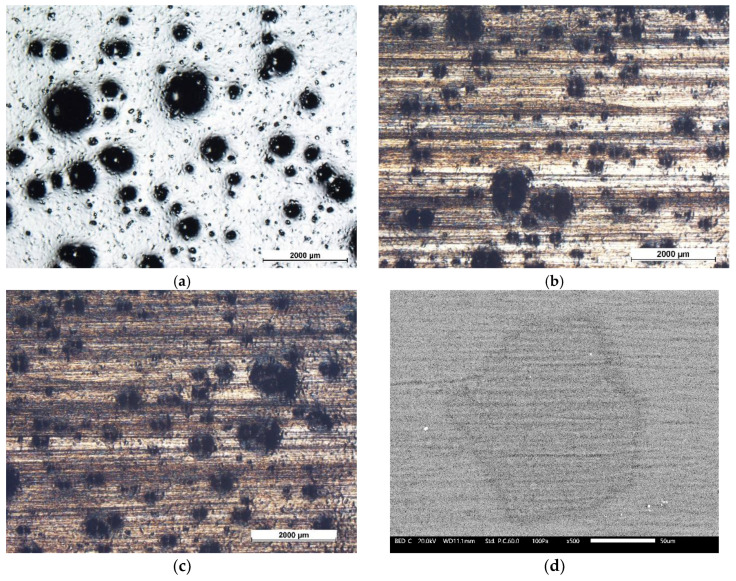
Optical stereomicroscope (**a**–**c**) and SEM (**d**) micrographs of sample SG surface morphology, taken (**a**) before the scrub test, (**b**) after 1000 cycles and (**c**,**d**) after 2000 cycles.

Figure 16a–c show the morphological evolution of the surface of the SG layer during the scrub test. In detail, the images reveal how the agglomerates of flakes effectively contrast the mechanical removal process due to the movement of the abrasive pad. Almost all of the surface of the sample is homogeneously covered by the pad grooves during the first 1000 cycles. This phenomenon is accentuated in the subsequent scrub cycles, till the end of the test. However, even after 2000 abrasion cycles, many superficial accumulations of graphene flakes are still evident. As a matter of fact, AFM analysis in scratching mode have evidenced high abrasion resistance offered by graphene-based fillers, especially in the form of agglomerates [60]. Graphene flakes, possessing high mechanical resistance, did not suffer particularly from the abrasive processes due to the scrub test. Consequently, the mass loss measured in the coatings containing the fillers, shown in Figure 14, is lower than the respective layers free of graphene-based flakes. Furthermore, the fillers show good abrasion resistance even as small agglomerates, or as single flakes, as revealed in Figure 16d. The agglomerate, slightly larger than 50 µm, underwent the abrasive process, but it was not removed due to the sliding of the pad, denoting high mechanical strength and good compatibility with the acrylic matrix of the coating. This result confirms the assumptions regarding the self-lubricating effect introduced by the fillers: as long as they are not completely removed from the bulk coating, the graphene-based flakes counteract the abrasion phenomena, also partially protecting the surrounding polymeric matrix. Ultimately, the coating CfGO-SG supplies excellent corrosion protection features, provided above all by the primer reinforced with fGO flakes, as well as high corrosion resistance, due to the graphene fillers contained in the spray top coat.

## 4. Conclusions

The use of graphene-based reinforcing fillers for the deposition of multifunctional acrylic coatings has been evaluated in this work. To take advantage of the different features of graphene, double layer coatings were deposited, employing two methods, such as cataphoresis and spray deposition process.

Graphene flakes, used in large quantities, exhibited a high tendency to agglomerate, introducing non negligible defects in the spray top-layers. Filler accumulation phenomena were observed by optical and electronic microscopy, together with a high level of porosity due to the spray deposition process.

Despite the deficiency of the top coat, the double layer coatings showed excellent behavior in an aggressive environment, due to the highly protective cataphoretic primer, reinforced by the optimized amount of fGO fillers. The fGO flakes slowed the solution absorption inside the coating, limiting the development of blisters following the creation of an artificial defect. Both layers, cataphoretic and sprayed, revealed good dry adhesion levels and excellent compatibility, as assessed by salt spray test and Cross Cut Test. The detachment of the coatings appeared to be almost negligible, well within the limits defined by the respective standards.

The electrochemical impedance measurements confirmed the excellent protective performance of the double layer coatings, which evidenced limited water adsorption phenomena owing to the effective barrier effect provided by the graphene-based flakes. After 500 h of immersion in the test solution, the double-layer coatings exhibited values of the impedance modulus 3–5 orders of magnitude higher than the results shown by the single layers, both cataphoretic and spray. The cataphoretic primer containing fGO flakes has been shown to play a key role in improving the corrosion protection properties of the acrylic matrix coating.

The large amount of filler added to the top spray coat, reaching the percolation level, significantly increased the conductivity of the acrylic matrix. Otherwise, the fGO flakes in the cataphoretic primer decreased the vertical conductivity of the coating, enlarging the electron and ion path length. Thus, the CfGO-SG sample is constituted by a highly insulating and protective primer and by a top-coat with high conductivity features.

Finally, the self-lubricating effect of graphene flakes and their good compatibility with the polymer matrix improved the wet abrasion resistance of the top coat. As a matter of fact, the scrub test revealed a good abrasion resistance of the SG layer, which positively influenced the protective performance of the double layer coatings C-SG and CfGO-SG.

The double layer sample CfGO-SG showed the best results in each analysis, thanks to the combined features of the primer and top coat, both reinforced by graphene-based fillers. Each layer possesses specific functions, depending on the type of filler with which it is reinforced. The cataphoretic primer provides good protective guarantees to the metal substrate, thanks to the optimized amount of fGO filler. Differently, the spray top coat plays the double role of conductive layer and anti-abrasion film, due to the high amount of graphene flakes which possess remarkable intrinsic features of conductivity and mechanical resistance.

Ultimately, this work illustrates a deposition procedure that exploits the chemical-physical features of graphene with the performance of the cataphoretic and spray deposition processes, for the creation of a smart, multifunctional coating.

## Figures and Tables

**Table 1:** Samples labelling, with the relative number of layers, deposition techniques and filler addition.

Sample	Number of Layers	Deposition Technique	Filler Addition
C	1	Cataphoresis	none
CfGO	1	Cataphoresis	fGO—0.2 wt %
S	1	Spray	none
SG	1	Spray	G—1 wt %
C-SG	2	Cataphoresis	none
		Spray	G—1 wt %
CfGO-SG	2	Cataphoresis	fGO—0.2 wt %
		Spray	G—1 wt %

**Table 2:** Coatings thickness measured with the thickness gauge.

Sample	First Layer [µm]	Second Layer [µm]	Total Coating [µm]
C	29.0 ± 0.8	/	29.0 ± 0.8
CfGO	28.6 ± 1.2	/	28.6 ± 1.2
S	55.0 ± 5.9	/	55.0 ± 5.9
SG	55.6 ± 5.2	/	55.6 ± 5.2
C-SG	30.6 ± 1.2	≈60	92.4 ± 2.8
CfGO-SG	30.9 ± 1.2	≈60	93.0 ± 3.2

**Table 3:** Pore resistance values of the six samples series.

Time [h]	Pore Resistance Rpore [Ω*cm^2^]					
	C	CfGO	S	SG	C-SG	CfGO-SG
3	6.85 × 10^5^	1.53 × 10^8^	2.36 × 10^6^	1.11 × 10^10^	7.15 × 10^9^	2.59 × 10^11^
15	2.81 × 10^5^	1.72 × 10^8^	2.95 × 10^6^	8.43 × 10^6^	5.96 × 10^9^	2.02 × 10^11^
48	3.45 × 10^5^	4.21 × 10^8^	3.26 × 10^6^	1.19 × 10^7^	2.14 × 10^9^	2.34 × 10^11^
170	5.29 × 10^4^	4.38 × 10^8^	3.43 × 10^6^	1.14 × 10^7^	1.57 × 10^9^	2.54 × 10^11^
340	2.25 × 10^4^	1.50 × 10^8^	7.50 × 10^6^	1.38 × 10^7^	1.28 × 10^9^	2.54 × 10^11^
500	2.45 × 10^4^	1.36 × 10^8^	6.84 × 10^6^	1.04 × 10^7^	1.87 × 10^9^	3.74 × 10^11^

**Table 4:** Coating capacitance values of the six samples series during the first 24 h of test.

Time [h]	Coating Capacitance C [F/cm^2^]					
	C	CfGO	S	SG	C-SG	CfGO-SG
0	1.69 × 10^−10^	1.86 × 10^−10^	1.38 × 10^−10^	2.86 × 10^−10^	5.88 × 10^−11^	5.93 × 10^−11^
4	2.57 × 10^−10^	1.95 × 10^−10^	3.78 × 10^−10^	3.70 × 10^−10^	6.74 × 10^−11^	8.03 × 10^−11^
8	3.40 × 10^−10^	1.99 × 10^−10^	5.28 × 10^−10^	4.62 × 10^−10^	7.32 × 10^−11^	8.03 × 10^−11^
12	4.43 × 10^−10^	2.83 × 10^−10^	6.46 × 10^−10^	5.55 × 10^−10^	8.08 × 10^−11^	8.08 × 10^−11^
16	5.45 × 10^−10^	3.26 × 10^−10^	8.35 × 10^−10^	6.35 × 10^−10^	8.22 × 10^−11^	8.13 × 10^−11^
20	7.07 × 10^−10^	3.38 × 10^−10^	1.55 × 10^−9^	7.65 × 10^−10^	8.71 × 10^−11^	8.15 × 10^−11^
24	1.50 × 10^−9^	3.40 × 10^−10^	3.20 × 10^−9^	1.10 × 10^−9^	9.17 × 10^−11^	8.17 × 10^−11^

**Table 5:** Volume resistivity measurements.

Sample	Voltage Applied V	Current Measured I	Volume Resistance R	Volume Resistivity ρ	Normalized Volume Resistivity
	[V]	[A]	[Ω]	[Ω*cm]	/
C	10	4.40 × 10^−10^	2.27 × 10^10^	1.71 × 10^12^	1.00
CfGO	10	7.20 × 10^−11^	1.39 × 10^11^	9.51 × 10^12^	5.56
S	10	4.30 × 10^−11^	2.33 × 10^11^	2.36 × 10^13^	13.79
SG	10	3.70 × 10^−5^	2.70 × 10^5^	2.55 × 10^7^	1.50 × 10^−5^
C-SG	10	1.80 × 10^−10^	5.56 × 10^10^	1.75 × 10^12^	1.03
CfGO-SG	10	3.45 × 10^−11^	2.90 × 10^11^	9.02 × 10^12^	5.28
